# Vaccine confidence and potential implications for new tuberculosis vaccines

**DOI:** 10.1186/s44263-025-00216-z

**Published:** 2025-10-31

**Authors:** Zsofia M. Hesketh, Rebecca A. Clark, Rupali Limaye, Puck T. Pelzer, Shaun Palmer, Richard G. White

**Affiliations:** 1 TB Modelling Group, TB Centre, Vaccine Centre, and Centre for Mathematical Modelling of Infectious Diseases, Department of Infectious Disease Epidemiology, LSHTM, London, UK; 2 Department of International Health, Johns Hopkins Bloomberg School of Public Health, Baltimore, MD USA; 3 International AIDS Vaccine Initiative (IAVI), 1013 NH Amsterdam, The Netherlands; 4 Stop TB Partnership Working Group On New Vaccines, New York, NY 10004 USA; 5 Gavi, The Vaccine Alliance, Geneva, Switzerland

**Keywords:** Tuberculosis, Novel vaccines, Vaccine confidence

## Abstract

**Background:**

A lack of general vaccine confidence has been identified as a potential barrier to the introduction of new tuberculosis (TB) vaccines. In the absence of TB-specific vaccine confidence surveys, analysis of general national vaccine confidence data can provide a useful proxy to determine where demand generation strategies may need to be focused ahead of future TB vaccine introductions.

**Methods:**

We analysed 2023 Vaccine Confidence Index (VCI) data from 18 of the 49 countries present on at least one of the three World Health Organisation (WHO) high TB burden lists, and together containing 65% of the global TB burden, to explore overall confidence in vaccines in high TB burden countries. Based on collected answers to three different statements, we categorised responses 1–2 as ‘positive’ (vaccine confident) and 3–4 as ‘negative’ (vaccine hesitant) and calculated a total vaccine confidence score using the mean proportion of positive responses across the three statements.

**Results:**

In 2023, over 80% of respondents in 14 of the 18 countries analysed, and over 60% of respondents in all 18 countries, believed that ‘vaccines are important for people of all ages’. India, accounting for around 30% of global TB cases, demonstrated confidence levels exceeding 90%, as did Vietnam, Ethiopia and Sierra Leone. South Africa, the country with the seventh highest TB burden (280,000 incident cases in 2023), Russia and Cameroon exhibited a relatively low vaccine confidence score of 75.5% or lower, signalling a potential area for concern. These countries may require focused awareness-raising and advocacy efforts prior to the rollout of new TB vaccines, though additional research on TB-specific confidence indicators is needed.

**Conclusions:**

This analysis underscores the importance of monitoring vaccine confidence levels to address emerging challenges to maintaining or bolstering the public’s trust in vaccination. Our findings could help determine which countries to prioritise for social mobilisation and demand generation efforts to boost vaccine confidence, and thus improve readiness for new TB vaccines.

**Supplementary Information:**

The online version contains supplementary material available at 10.1186/s44263-025-00216-z.

## Background

TB continues to be one of the leading infectious causes of morbidity and mortality worldwide, with an estimated 10.8 million people falling ill (corresponding to 134 incident cases/100,000 population) and 1.25 million dying from the disease in 2023 [1]. For the 2021–2025 period, the WHO has established three lists of 30 countries to identify those settings with the highest burden of TB, TB/human immunodeficiency virus (HIV) co-infection, and multidrug- or rifampicin-resistant TB (MDR/RR-TB) respectively. Whilst the lists are distinct, there is some overlap in the countries present within them. In total, 49 countries belong to at least one of these three lists, including 10 countries (China, DR Congo, India, Indonesia, Mozambique, Myanmar, Nigeria, Philippines, South Africa and Zambia) that are part of all three [2].

The Bacillus Calmette-Guérin (BCG) vaccine, which was developed in 1921, is currently the only licensed TB vaccine in use and is recommended in countries with high TB burden [3]. When administered neonatally, BCG provides protection against disseminated and meningeal TB for around 10 years [3, 4]. However, its efficacy against pulmonary TB in adults and adolescents is variable and more limited [5–8], leading to growing efforts to develop novel vaccines in recent decades. There are currently 15 novel TB vaccine candidates being evaluated in different stages of clinical development [9]. Six of these novel vaccines, intended for use in adults and adolescents, are in late-stage clinical trials at the time of writing: BCG (as a travel vaccine), GamTBvac, M72/AS01_E_, MIP, MTBVAC and VMP1002 [9–11]. It is therefore important to prepare for implementation ahead of potential licensure to minimise delay.

Low confidence in vaccines in general has been identified as an anticipated barrier to introducing new TB vaccines, with the potential to delay and/or reduce uptake [11, 12]. It is defined here as a sliding scale of attitudes and feelings that increases the likelihood of accepting and/or encouraging vaccination on one end, and of hesitating and/or refusing to vaccinate on the other. Vaccine refusals are contributing to an increasing number of vaccine-preventable disease outbreaks globally, such as measles. For this reason, vaccine hesitancy, the low end of the confidence scale defined as a ‘delay in acceptance or refusal of vaccination despite availability of vaccination services’, was named by the WHO as one of the top ten threats to global health in 2019 [13, 14]. Existing data on COVID-19, human papillomavirus (HPV) and polio vaccine confidence across more than 20 countries suggests that individuals’ trust in the government, pharmaceutical industry and societal structures is associated with the intention to get vaccinated [15, 16]. One of the studies, which sampled responses from Ghana, Kenya, Nigeria, South Africa, Tanzania and Uganda, found these associations to remain consistently strong across each of the three vaccines, suggesting that measuring trust may be a useful indicator of the intention to get vaccinated. Successful introduction of new TB vaccines may require an understanding of not just vaccine confidence specifically, but of broader societal trust levels to allow careful planning around vaccine hesitancy mitigation.

In the absence of detailed national TB-specific vaccine confidence data, we analysed the most up-to-date 2023 vaccine confidence data from the VCI [17] for countries appearing on one of WHO’s high TB burden lists [2]. Using these data, we calculated overall 2023 vaccine confidence scores for the high TB burden countries in the dataset, as well as disaggregated scores for each of the three individual survey variables. We used the results of these two analyses as a proxy for TB vaccine confidence, based on evidence that general attitudes towards vaccine importance, safety and effectiveness are strong predictors of acceptance of both established and novel vaccines. We then inferred where low vaccine confidence levels may hinder new TB vaccine uptake in the future, and where demand generation, advocacy or other context-specific strategies may therefore need to be focused.

## Methods

To estimate vaccine confidence, we used data gathered from the Vaccine Confidence Project’s VCI [17]. Data collection began in 2015 [18], conducted in collaboration with ORB International (Gallup International) and the Wellcome Global Monitor, and has since grown to a total sample of over 430,000 across 156 countries, though the exact set of countries sampled differs from year to year. All data collections were conducted with samples nationally representative of the general population and included participants’ age group (18–24, 25–34, 35–44, 45–54, 55+) and sex. For certain countries and in certain years, participants’ religion and maximum educational level are also recorded.

We extracted data from 2023, a sample which constitutes a total of 70,781 responses from a total of 70 countries, 18 of which (Brazil, Cameroon, DR Congo, Ethiopia, India, Indonesia, Kenya, Liberia, Nigeria, Pakistan, Philippines, Russian Federation, Sierra Leone, South Africa, Thailand, Uganda, Ukraine, Vietnam) appear on at least one of the WHO’s three high TB burden lists [2]. We then analysed responses to three VCI statements, ‘*Overall, I think vaccines are important for people of all ages*’, ‘*Overall, I think vaccines are safe*’ and ‘*Overall, I think vaccines are effective*’, which were provided on a numeric Likert scale from strongly agree (value = 1) to strongly disagree (value = 4), with intermediate values for ‘tend to agree’ (2) and ‘tend to disagree’ (3). We did not include responses to a fourth statement in the dataset, ‘*Overall, I think vaccines are compatible with my religious, personal or philosophical beliefs*’, for ease of comparability of our results with Figueiredo et al.’s earlier analysis.

For responses from these 18 high TB burden countries, we categorised values 1–2 as ‘positive’ (vaccine confident) and 3–4 as ‘negative’ (vaccine hesitant) and calculated a total vaccine confidence score using the mean proportion of positive responses across the three statements. Prior to data processing and analysis, we excluded all ‘Don’t Know’ responses (making up 2.4%, 3.7% and 3.5% of responses to the ‘important’, ‘safe’ and ‘effective’ statements respectively) as we considered these missing values.

**Fig. 1:**
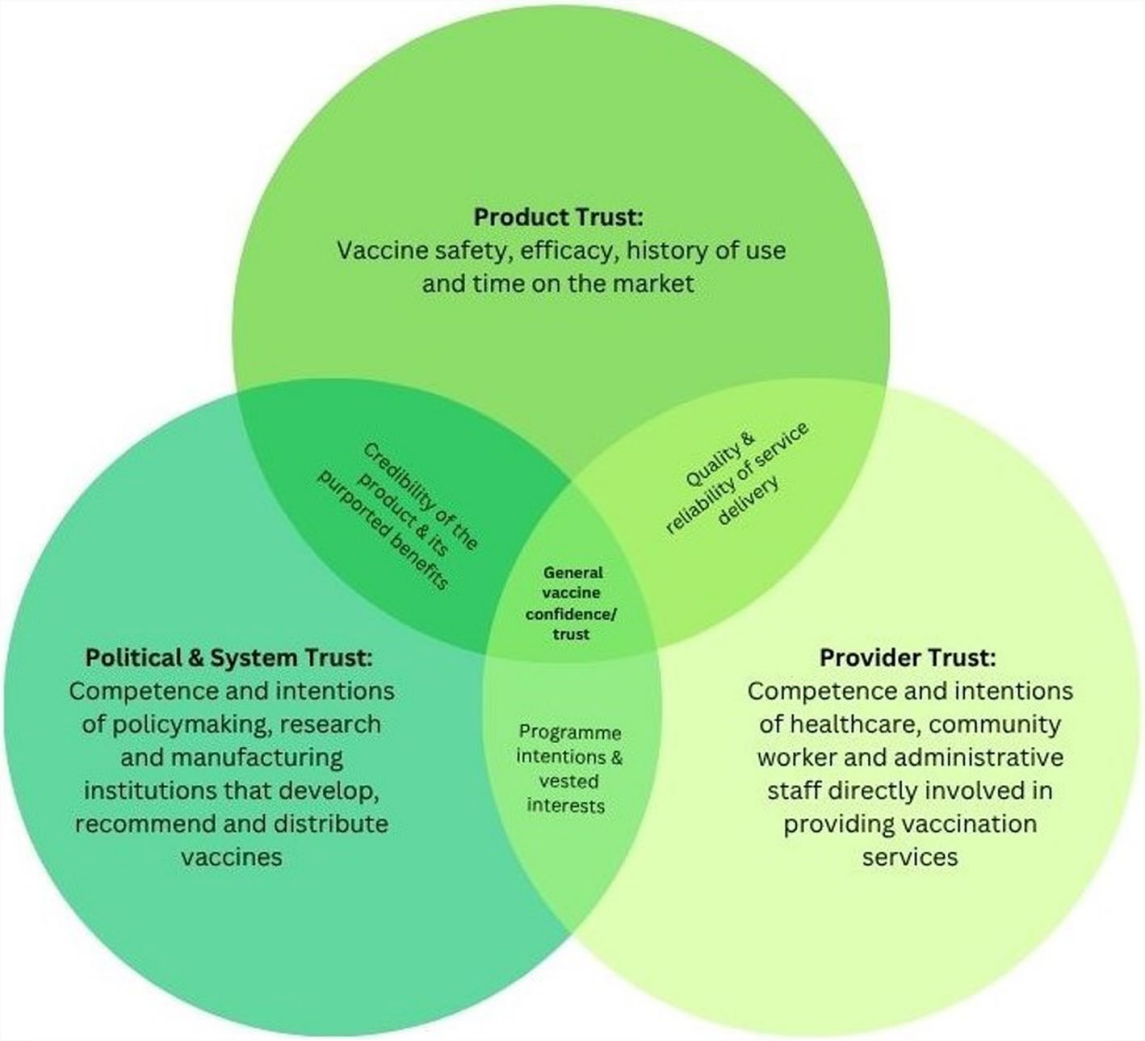
A visualisation of the trust relationship as the general framework for vaccine confidence. We conceptualised confidence as being formed of three different trust dimensions: relating to the product itself (its perceived safety, efficacy and historic use), relating to the healthcare providers in direct contact with the individual or community and relating to the broader ‘system’ made up of political, economic, pharmaceutical and research institutions. Figure adapted from the vaccine trust dimensions and graphical representations elaborated by Larson et al. [19]

Confidence in vaccines is recognised as a multidimensional construct encompassing perceived importance, safety and effectiveness, which have been identified by prior studies as key drivers of public confidence in vaccines and which collectively shape public acceptance of both established and less familiar products [13, 16]. We therefore assumed that generating a general vaccine confidence score based on the mean proportion of positive responses to these three statements would be an appropriate indicator of confidence in a new TB vaccine, serving as a proxy to gauge the likelihood of seeking or accepting its use. This is especially relevant in contexts where the new vaccine is perceived as emerging from the same institutions or systems that administer established immunisation programmes. As each of the three survey statements probes into these different yet interlinked dimensions of confidence—such as the perceived health-protective effects of vaccines as well as the competence and motives of medical, pharmaceutical and governmental institutions engaged in the vaccination space—they may provide valuable initial insights for approximating vaccine confidence, particularly in relation to newly developed products like novel TB vaccines which can elicit stronger reactions and suspicions on these dimensions (Fig. 1) [18, 19].

For this reason, we also calculated individual confidence scores per country as the proportion of positive responses to each of the three statements. Comparing the disaggregated scores to the overall score was assumed to help build a fuller picture of the climate of public opinion and the dimensions of trust that may be more or less prone to vaccine hesitancy in a given country.

Finally, to understand whether any age and sex-associated variation was present in the sample, we calculated an overall confidence score by sex and age group for each of the 18 countries analysed, using the same method described. The complete analytic code is available on GitHub [20]. Corresponding WHO estimates for TB burden (as of November 2024) were also recorded for each of the 18 countries analysed, expressed as both the total number of incident TB cases and the number per 100,000 population [21].

## Results

Vaccine confidence data was available in 2023 for 18 of the 49 countries on one of the WHO lists containing 65% of the global TB burden [21]. The total number of respondents from these 18 countries, after exclusion of ‘Don’t Know’ responses, was 18,558 for the statement ‘*Overall, I think vaccines are important*’, 18,324 for the statement ‘*Overall, I think vaccines are safe*’ and 18,350 for the statement ‘*Vaccines are effective*’. The age of respondents ranged from 18 to 55+ years, grouped across five discrete categories (18–24, 25–34, 35–44, 45–54, 55+) with most respondents (around 30%) aged 25–34 years. On average, responses from each country came from approximately 1000 individuals. The VCI survey, similar to those collected several years ago, was weighted by sex and age according to national distributions and had near-equal sex representation for most countries [13].

Looking at the overall vaccine confidence scores (Table 1), at least 80% of respondents across 14/18 countries judged vaccines positively. Vietnam had the highest combined score among the countries we analysed with a confidence score of 98.5%, followed by Ethiopia (95.5%), India (91.8%) and Sierra Leone (90.6%). Among the top five highest TB burden countries we analysed [1, 21] (India, Indonesia, the Philippines, Pakistan and Nigeria), vaccine confidence was between 83.8% and 91.8%, with India—the country with the highest absolute number of incident TB cases, making up 38.9% of the TB burden [21] among the list of countries we analysed—exhibiting the highest total vaccine confidence based on the combined score. The four countries with the lowest overall confidence score were Cameroon (63.0%), Ukraine (66.2%), Russia (70.5%) and South Africa (75.5%).

**Table 1:** Combined vaccine confidence scores (percent positive) by country. Calculated using responses to the three statements (i) ‘*Overall, I think vaccines are important*’, (ii) ‘*Overall, I think vaccines are safe*’ and (iii) ‘*Overall, I think vaccines are effective*’ and ordered by highest to lowest number of incident TB cases in 2023. Data source: WHO TB Burden Estimates as of November 2024, https://www.who.int/teams/global-tuberculosis-programme/data [21]

Country	High TB burden list in which present	Number of incident TB cases in 2023 [21]	Number of incident TB cases per 100,000 [21]	Number of individuals surveyed in 2023	Combined vaccine confidence score for 2023 (% positive)
India	TB, HIV-TB, MDR/RR-TB	2,800,000	195	1177	91.8
Indonesia	TB, HIV-TB, MDR/RR-TB	1,090,000	387	1000	84.3
Philippines	TB, HIV-TB, MDR/RR-TB	739,000	643	1000	84.8
Pakistan	TB, MDR/RR-TB	686,000	277	1065	83.8
Nigeria	TB, HIV-TB, MDR/RR-TB	499,000	219	1032	89.1
DR Congo	TB, HIV-TB, MDR/RR-TB	334,000	316	1030	83.2
South Africa	TB, HIV-TB, MDR/RR-TB	280,000	427	1000	75.5
Vietnam	TB, MDR/RR-TB	182,000	182	1000	98.5
Ethiopia	TB, HIV-TB	188,000	146	1184	95.5
Kenya	TB, HIV-TB	124,000	223	1083	89.3
Thailand	TB, HIV-TB	113,000	157	1041	87.2
Brazil	TB, HIV-TB	103,000	49	1005	88.5
Uganda	TB, HIV-TB	96,000	198	1050	88.9
Russia	HIV-TB, MDR/RR-TB	55,000	38	1012	70.5
Cameroon	HIV-TB	43,000	150	1100	63.0
Ukraine	MDR/RR-TB	42,000	112	1018	66.2
Sierra Leone	TB	24,000	283	1039	90.6
Liberia	TB, HIV-TB	17,000	308	1086	87.9

Drilling down into overall confidence scores by sex, females judged vaccines more positively than males in 12 of the 18 countries in our dataset. However, the difference between female and male confidence scores did not exceed 5.7% and generally showed no more than a 2–3% difference—except in one country, Cameroon, where the overall vaccine confidence score of male respondents was 11.1% lower than that of female respondents. The range of confidence scores observed among the five age groups, particularly with regard to the highest and the lowest-scoring age group and the difference between them, varied more substantially from country to country. In 50% of the countries in our dataset, it was the 55+ age group that reported the most favourable overall view of vaccines, followed by the 18–24 age group (in four countries: Cameroon, India, Sierra Leone, Ukraine), both the 25–34 and 45–54 age groups (each in two countries, Brazil and South Africa and Ethiopia and Thailand respectively) and the 35–44 age group in one country, Liberia. Conversely, the age group that scored the lowest was most frequently the 25–34 cohort (in six countries), followed by the 18–24 cohort (in five countries). The 55+ age group was only the lowest scoring in Cameroon. The percentage difference between the highest- and the lowest-scoring age groups ranged between just 1.2% in Vietnam and 15.2% in Nigeria, and in four more countries—Ukraine, Pakistan, Uganda and Cameroon—a difference of more than 10% was observed between the most and least confident age cohorts. Detailed data tables for both the sex-disaggregated and age group-disaggregated confidence scores can be found in Supplementary Material 1: Table S1 and Supplementary Material 1: Table S2, and individual country charts visualising this data can be found in Supplementary Material 2: Fig. S1 and Supplementary Material 2: Fig. S2 respectively.

Concerning the results of the disaggregated analysis of confidence score per individual statement (Table 2), we found that all countries except Vietnam scored highest on the ‘vaccines are important’ statement. The confidence scores for each of the three questions were within 5% of each other in only seven countries, namely India, Pakistan, Nigeria, South Africa, Vietnam, Ethiopia and Cameroon, together representing 39% of the incident TB cases [21] among the countries we analysed. Across the sample, the mean percentage difference between the ‘vaccines are important’ statement and the ‘vaccines are safe’ statement was approximately 5.1%, compared to the mean percentage difference with the ‘vaccines are effective’ statement coming to 4.8%.

**Table 2:** Disaggregated 2023 confidence score per country per survey statement. Ordered by highest to lowest number of incident TB cases recorded in 2023. Data source: WHO TB Burden Estimates as of November 2024, https://www.who.int/teams/global-tuberculosis-programme/data [21]

Country	Number of incident TB cases in 2023 [21]	Number of incident TB cases per 100,000 [21]	Percent positive ‘*Vaccines are important*’	Percent positive ‘*Vaccines are safe*’	Percent positive ‘*Vaccines are effective*’
India	2,800,000	195	93.2	90.9	91.3
Indonesia	1,090,000	387	87.5	83.0	82.4
Philippines	739,000	643	89.1	81.5	83.8
Pakistan	686,000	277	84.4	84.2	82.9
Nigeria	499,000	219	89.7	88.9	88.7
DR Congo	334,000	316	86.8	79.6	83.3
South Africa	280,000	427	75.7	75.2	75.6
Vietnam	182,000	182	98.9	97.3	99.4
Ethiopia	188,000	146	97.0	94.1	95.4
Kenya	124,000	223	93.7	89.3	84.8
Thailand	113,000	157	93.3	83.3	85.1
Brazil	103,000	49	92.2	85.7	87.7
Uganda	96,000	198	94.1	86.5	86.2
Russia	55,000	38	78.5	61.1	71.9
Cameroon	43,000	150	65.1	61.7	62.2
Ukraine	42,000	112	71.5	60.9	66.3
Sierra Leone	24,000	283	94.1	91.6	86.0
Liberia	17,000	308	93.1	90.8	79.8

**Fig. 2:**
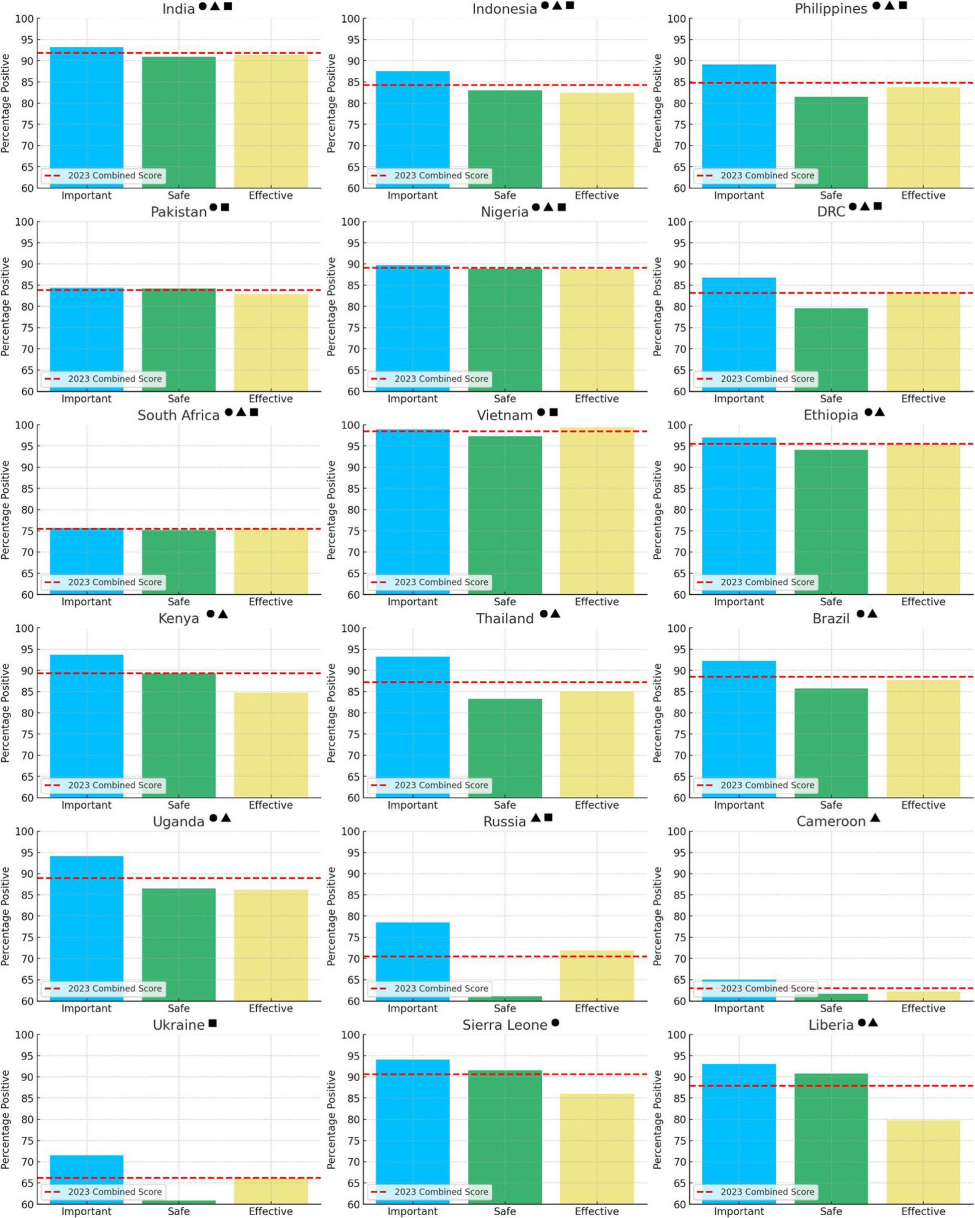
Country by country statement-disaggregated and combined vaccine confidence score. For each of the 18 high TB burden countries surveyed in 2023, three bar charts representing the % positive responses for each of the three ‘Vaccines are important’, ‘Vaccines are safe’ and ‘Vaccines are effective’ statements, as related to the overall vaccine confidence score. Key: ● = country is part of the high TB burden list, ▲ = country is part of the high HIV-TB burden list, ■ = country is part of the high MDR/RR-TB burden list

A visual comparison of country-by-country disaggregated scores (Fig. 2) illustrates that several countries, namely Thailand, Russia, Ukraine and Liberia, exhibit notable discrepancies between the different dimensions of vaccine confidence as measured by comparing the percentage positive response to the three statements on vaccine importance, safety and efficacy. Russia, Ukraine and Thailand exhibit a 17.4%, 10.6% and 10% drop respectively between respondents’ assessment of vaccine importance and vaccine safety. Conversely, Liberia results show the confidence score surrounding vaccine effectiveness being 13.3% lower than the confidence score on the importance dimension.

## Discussion

We analysed data from 18 of the 49 countries present on at least one of the three WHO high TB burden lists [2], containing 65% of the global TB burden [21], to explore overall confidence in vaccines in high TB burden countries. The dataset reveals some regional variation in vaccine confidence, with South and Southeast Asian countries reporting some of the highest confidence levels. African countries exhibited broader variability both across and within regions, with Ethiopia and Sierra Leone judging vaccines more positively than South Africa and Cameroon. Confidence was generally lower in Eastern Europe, suggesting the influence of socio-political and other regional factors.

We assumed that a combined vaccine confidence score, based on the mean proportion of positive responses to the three statements ‘*Overall, vaccines are important for people of all ages*’, ‘*Overall, vaccines are safe*’ and ‘*Overall, vaccines are effective*’, would be an appropriate indicator of public confidence in a new TB vaccine. This is because the three statements each speak to different concepts and beliefs that underlie the extent to which individuals place trust in vaccination, including whether vaccines bring overall health benefits and should be regarded as a public good; whether vaccines are useful to administer in older age groups beyond infancy; and whether the scientific and governmental institutions developing, recommending and delivering vaccines are reliable and acting in the best interests of the population [18, 19].

 Looking at country-specific results, over 80% of respondents in 14 countries and over 60% of respondents in all 18 countries believed that ‘vaccines are important for people of all ages’. India, accounting for around 30% of global TB cases [1, 21], demonstrated confidence levels exceeding 90%, as did Vietnam, Ethiopia and Sierra Leone. South Africa, the country with the seventh highest TB burden (280,000 incident cases in 2023) [21], as well as Russia, Ukraine and Cameroon exhibited a relatively low vaccine confidence score of 75.5% or lower, signalling a potential area for concern. These countries may require focused awareness-raising and advocacy efforts prior to the rollout of new TB vaccines, though additional research on TB-specific confidence indicators is required.

Of the 18 countries we analysed, Cameroon’s 2023 confidence score was lowest (63.0%), but this may pose a relatively modest concern given its low share of the global TB burden (44,000 incident TB cases, corresponding to 0.61% of the burden figure across the countries we analysed) and thus require lower prioritisation for novel TB vaccine access. Nonetheless, potential hesitancy-driving events may be important to monitor going forward. In recent years in Cameroon, low rates of COVID-19 vaccine confidence and medical mistrust have been linked to the consumption of social media-associated misinformation [22], an effect which may continue to contribute to decreasing vaccine confidence in the future. The history of vaccine hesitancy in Cameroon and neighbouring Central African countries has also been posited to have deeper, more intractable roots, beginning as a response to French colonial medical campaigns in the first half of the twentieth century [23]. Forced medical examinations may have generated a latent sense of mistrust in similar interventions to this day, making careful and locally grounded social mobilisation important areas of focus ahead of novel TB vaccine introduction.

Demand-generation and sensitisation efforts could also be targeted to Ukraine and Russia, the next lowest-scoring countries which have a high burden of MDR/RR-TB and are additionally at risk of heightened TB infection transmission as a result of ongoing conflict [24]. Importantly, we also identified South Africa as a country that would benefit from concerted efforts given that it is the seventh highest burden country in our dataset (containing 3.9% of the total TB burden across the 18 countries analysed) yet has a relatively low overall vaccine confidence score of 75.5%. This figure is lower than the 82.5% vaccine confidence reported in Unfried and Priebe’s analysis [16], though measured via two different questions: intention to get vaccinated and intention to recommend vaccination to others.

Evidence-based approaches to demand generation could include drawing on risk communication strategies, such as leveraging trusted community members to convey messages with enhanced credibility and the facilitation of two-way, open exchange that do not shy away from ‘awkward conversations about politics, power, and interests, as well as the broader structural injustices people face’ [25]. In South Africa itself, a study conducted by Eh!Woza—a local public engagement, youth education and advocacy initiative—has identified actionable TB vaccine-specific advocacy and acceptance measures [26]. These include speaking to people in a language they understand, and—given that TB remains highly stigmatised—deploying these advocacy and popular education efforts at scale in places where they feel comfortable (e.g. churches, schools). Strategies targeting broader systemic changes may include ensuring that health delivery centres are clean, functional and well-led, as ‘the attitude of staff… quality of leadership and [of] the service’ all contribute to people’s trust and willingness to receive care [26].

We also undertook demographic subgroup analyses by examining the differences in vaccine confidence by sex and age group, with findings suggesting the existence of variability across countries. Whilst females expressed more positive views towards vaccines than males in 12 of the 18 countries, this difference was relatively modest, rarely exceeding 5.7%, and often limited to only two to three percentage points. An exception was observed in Cameroon, where male respondents exhibited a substantially lower vaccine confidence score of 58.1% compared to 69.2% in female (representing an 11.1% difference). This suggests that specific local cultural or socio-economic factors may be at play in influencing vaccine perceptions, an inference supported by the existing literature, which presents conflicting findings regarding gender differences in vaccine confidence and thereby implies that discrepancies may be highly context-dependent [16, 27, 28].

Regarding the relationship between age group and vaccine confidence, the data revealed a more diverse pattern across countries. In nine of the 18 countries surveyed, the 55+ age group judged vaccines most favourably, followed by the 18–24 age group in four countries (Cameroon, India, Sierra Leone, Ukraine). Younger cohorts (18–24 and 25–34) were the least confident in 11 of the 18 countries, potentially owing to greater exposure to misinformation via the consumption of online media, and the range between of scores within each country was between a modest 1.2% in Vietnam to a more substantial 15.2% in Nigeria, followed by Ukraine (13%), Pakistan (12.4%), Uganda (13.3%) and Cameroon (13.3%). In Cameroon, for example, the historical legacy of harmful and non-consensual colonial medical practices may help explain why older age groups, who may have direct or familial recollections of these experiences, report lower vaccine confidence compared to younger cohorts [23]. These variations highlight the importance of context when interpreting vaccine confidence data and the potential need for tailored public health messaging strategies that address the specific concerns of different age cohorts. However, data must be interpreted carefully, as the number of respondents can differ substantially within each cohort and generally follows the pattern of each country’s stage in the demographic transition model. As reported in Supplementary Material 1: Table S2, dataset countries from the Africa and South Asia regions with high birth rates and younger populations generally had a much smaller sample of respondents from older age cohorts, and vice versa for countries like Russia, Ukraine, Thailand and Vietnam, whose birth rates are lower and average population age higher.

Additional analyses could be useful to understand the cause of the large discrepancies observed between some countries’ disaggregated scores of different dimensions of vaccine confidence. Namely, the respective 17.4%, 10.6% and 10% difference between Russia, Ukraine and Thailand’s vaccine importance and vaccine safety score signals a potential mistrust of the vaccine development process and of new vaccine products without an established safety profile. Meanwhile, the 13.3% difference between vaccine importance and vaccine effectiveness scores in Liberia may point to insufficient or unclear information about vaccines’ impact, despite general knowledge of the public that receiving them is good, customary, or better than no intervention. Overall, these discrepancies highlight that whilst there is some level of trust in the importance of vaccines, substantial concerns about safety or effectiveness could undermine their overall uptake and resulting coverage, potentially including that of future TB vaccines.

Although TB vaccine-specific confidence data were unavailable at the time of writing, our results can be compared to those of other studies of general vaccine confidence. Figueiredo et al.’s analysis of 2015–2019 VCI data [13] includes some overlap with the countries in our dataset and assessed the same three confidence dimensions (safety, efficacy and importance). In 2019, among countries overlapping with this earlier analysis, Uganda, Liberia and India ranked highest in the proportion of respondents agreeing that vaccines are safe; Liberia, Ethiopia and India ranked highest in the proportion of respondents agreeing that vaccines are important; and India, Ethiopia and Kenya ranked highest in the proportion of respondents agreeing that vaccines are effective [13]. These earlier results largely match our current analysis, in which each of these countries, with the exception of Kenya, has a confidence score above 90% on the corresponding dimension. Respondents from India have been shown to be consistently confident in vaccines even when measured in different contexts and using different methodologies. During the COVID-19 pandemic, which saw a rise in vaccine misinformation and subsequent hesitancy, over 95% of respondents from India reported willingness to take a COVID-19 vaccine once available if it were recommended by a doctor or employer [15]. This continuity of high vaccine confidence in India, as reflected in our results as well as earlier analyses, is heartening given its status as the highest burden country for TB, making it one of the first in line for novel TB vaccine access.

Whilst we assumed that combined responses to ‘*Overall, vaccines are important for people of all ages*’, ‘*Overall, vaccines are safe*’ and ‘*Overall, vaccines are effective*’ would be an appropriate indicator of the confidence in a new TB vaccine, we acknowledge several limitations to this approach. For example, using the combined score we calculated could obscure key differences in public perceptions of the overall importance of vaccination compared to confidence in the perceived safety and efficacy of vaccines that have recently been or will soon be added to a country’s vaccination schedule. As a novel TB vaccine would fall into this latter category, understanding these potential differences could inform targeted awareness-raising and demand generation initiatives. COVID-19 vaccine rollout provides a relevant precedent for this need for nuance, as scepticism arose towards novel products that had been developed more recently, rapidly, or using unfamiliar technologies, despite high levels of trust in vaccination being reported more generally [29–31].

As vaccine confidence is founded on trust [13, 16, 18, 19], which is in turn facilitated by clear communication and information-sharing, newly developed vaccines may face confidence issues when first rolled out as they are less well-known or commonly used [30]. To capture these distinctions, it could be beneficial to update and expand the list of questions used to collect vaccine confidence data, incorporating statements measuring the willingness or intent of participants to be vaccinated with novel vaccines versus established vaccines.

Beyond differentiating between established and novel vaccines, one principal limitation of our approach is the absence of TB-specific indicators in the available dataset, such as intention to vaccinate based on perceived risk of TB, in the context of TB-associated stigma, or as a choice among other TB prevention modalities [11]. These vaccine-specific indicators may influence confidence in a new TB vaccine and need to be considered in follow-up research to facilitate a more accurate understanding of underlying dynamics. A set of TB-tailored questions that probe into TB stigma, TB burden, perceived TB risk and other contextual factors could be developed separately to the VCI and integrated into other ongoing survey and stakeholder consultation efforts. These initiatives would ideally be targeted to high TB burden countries only and could, for example, form part of their early planning for potential novel TB vaccine introduction. Targeted data collection is especially important given that we currently lack data for the other 31 high TB burden countries [2], which limits our ability to generalise findings beyond the contexts where this data is available. Without this, predictions of, and optimal strategies to strengthen, TB vaccine confidence in other high-burden, high-urgency contexts remain speculative.

Another related limitation is that individual interpretations of the terms ‘important’, ‘safe’ and ‘effective’ may vary; responses may not accurately reflect true confidence levels which could reduce the generalisability of the findings. Whilst it is unrealistic to completely eliminate subjective interpretation, it is possible to reduce it by developing more standardised confidence survey formats that include specific definitions of key terms. As well, our omission of responses to a fourth statement on vaccines’ compatibility with religious, personal and philosophical beliefs means that there is missing data that could be subject to further analysis. Including a fourth dimension of compatibility with beliefs could add granularity to our interpretation of the disaggregated scores but also impact a country’s overall confidence score, a potential effect that is at present obscured by its exclusion.

Finally, whilst novel TB vaccine candidates target both adolescents and adults, the VCI dataset [17] exclusively surveyed adults aged 18 and older, thereby leaving a blind spot around younger target groups’ confidence levels. This information gap may not be overly consequential, as it is often adult caregivers and guardians that make vaccination decisions on behalf of their children up until they reach adulthood; in this sense, surveying adults may provide insights not only into their own acceptance of vaccination but also their willingness to promote it for others, such as teenage relatives [32]. Nonetheless, to comprehensively understand confidence levels in adolescent target groups, including respondents under 18 years of age would be a valuable expansion of survey samples [16]. Such data would capture the attitudes of young people making their own health decisions, such as those living without parental care, as well as forecasting the attitudes of the future adult cohort by the time a novel TB vaccine is rolled out.

## Conclusions

With 15 novel TB vaccines in the development pipeline, six of which are already in phase 3 clinical trials, new TB vaccines that prevent pulmonary TB in adolescents and adults could be available within the next decade [9–11]. Our analysis underscores the importance of monitoring vaccine confidence levels to maintain or bolster the public’s trust in vaccination, particularly for high-burden countries that would benefit most from earlier programme launches. Additional data are still needed to fully understand the degree of TB vaccine-specific confidence in high TB burden countries, which may shed light on the impact of disease-specific stigma as well as the attitudes of individual target age groups. As we await these additional data, our findings could begin to help determine which countries to prioritise for social mobilisation and demand generation efforts to boost vaccine confidence, and thus improve readiness for introducing new TB vaccines.

## Supplementary Information

Supplementary Material 1. Sex- and age-disaggregated vaccine confidence data tables. Contains sex- (Table S1) and age group- (Table S2) disaggregated 2023 vaccine confidence scores from the 18 high TB burden countries, including overall and subgroup confidence scores for the statements ‘Vaccines are important’, ‘Vaccines are safe’ and ‘Vaccines are effective.

Supplementary Material 2. Sex- and age-disaggregated vaccine confidence charts by country. Bar charts showing overall vaccine confidence scores disaggregated by sex (Fig. S1) and by age group (Fig. S2) for each country.

## Data Availability

The analytic code for both the main analysis and the sex- and age group-disaggregated analysis is publicly available from https://github.com/zmhesketh/2023_TBVaccine_Confidence/tree/main [20]. Country-by-country data on the latest number of incident TB cases are publicly available and downloadable from the WHO website (https://www.who.int/teams/global-tuberculosis-programme/data) [21]. Vaccine confidence data from the VCI are publicly available on the VCP website, covering general 2015-2024 survey data results (https://www.vaccineconfidence.org/vci/data-and-methodology/) [17]. The complete 2023 VCI dataset used in this manuscript, which includes additional raw variables, is not publicly available due to ongoing processing by members of the VCP. For this reason, it is also not directly shareable by the authors as it belongs to the VCP. To obtain the detailed 2023 dataset, we made a request using the VCP contact form (https://www.vaccineconfidence.org/contact-us/) and via email to Prof. Heidi Larson (heidi.larson@lshtm.ac.uk).

## Abbreviations

BCG: Bacillus Calmette-Guérin
HIV: Human immunodeficiency virus
HPV: Human papillomavirus
TB: Tuberculosis
MDR/RR-TB: Multidrug/rifampicin-resistant tuberculosis
VCI: Vaccine Confidence Index
VCP: Vaccine Confidence Project
