# Supplementary material for: Vaccine confidence and potential implications for new tuberculosis vaccines
Source: BMC Glob Public Health. 2025 Oct 31;3:96. doi: 10.1186/s44263-025-00216-z (PMC12577312; doi:10.1186/s44263-025-00216-z)
Supplement: Supplementary file 2 — Supplementary Material 2. Sex- and age-disaggregated vaccine confidence charts by country. Bar charts showing overall vaccine confidence scores disaggregated by sex (Fig. S1) and by age group (Fig. S2) for each country. [file 44263_2025_216_MOESM2_ESM.pdf]

Additional File 2: Vaccine confidence and potential implications for new tuberculosis vaccines

Zsofia M. Hesketh, Rebecca A. Clark, Rupali Limaye, Puck T. Pelzer, Shaun Palmer, Richard G. White

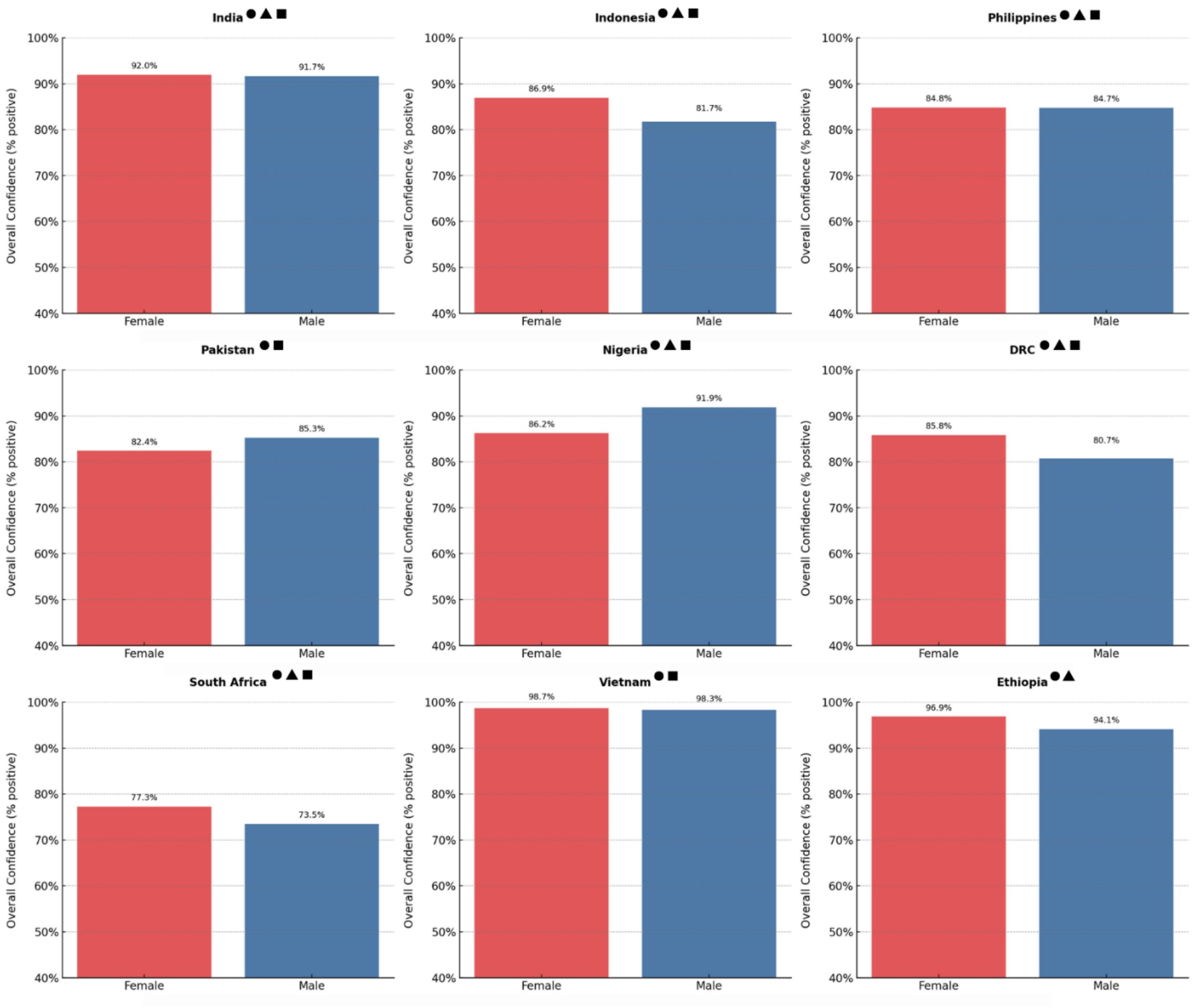

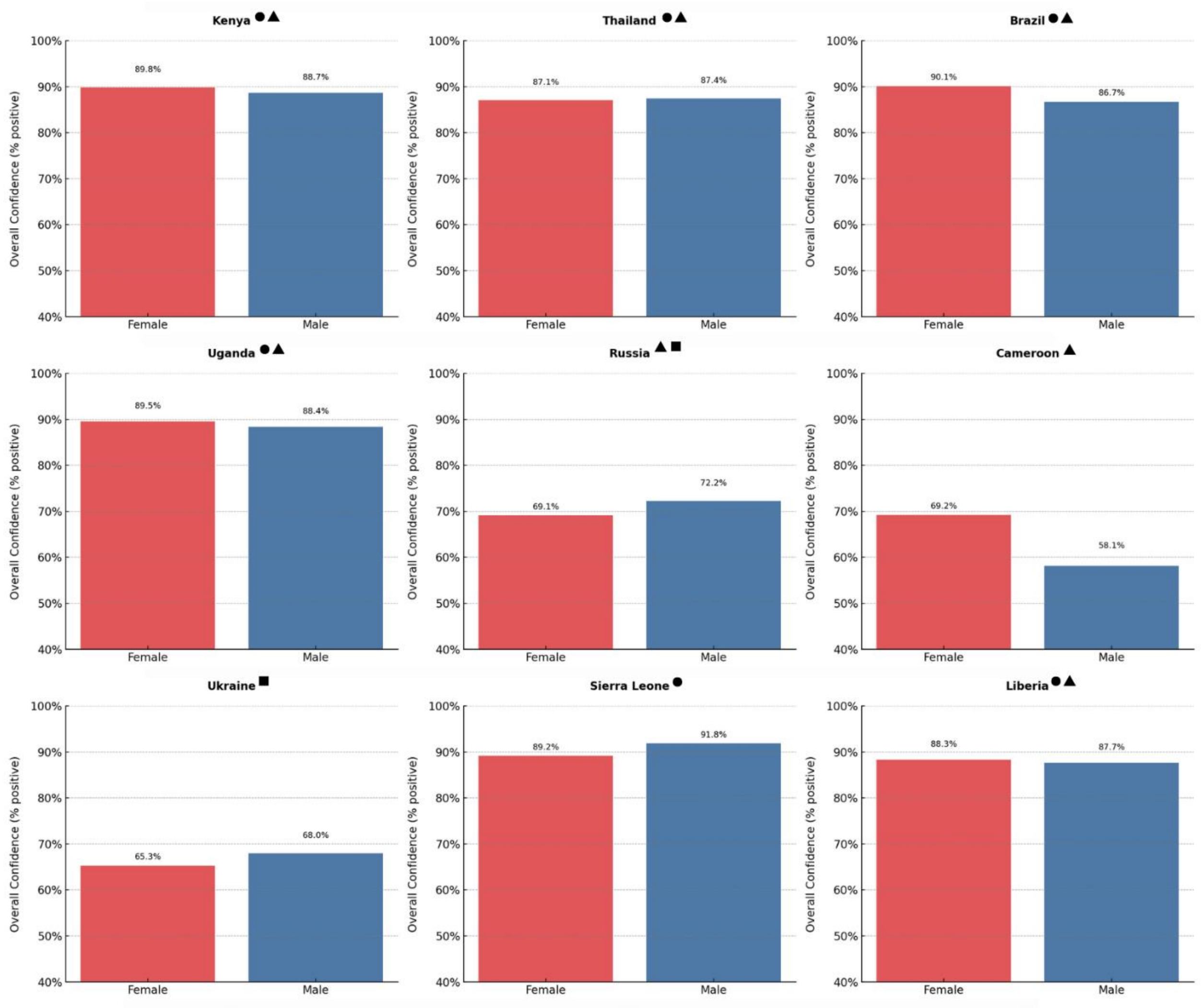

Figure S1. Sex-disaggregated overall vaccine confidence score by country. For each of the 18 high TB burden countries surveyed in 2023, two bar charts representing the overall female and male vaccine confidence scores calculated as the average % positive responses to the three statements "Vaccines are important", "Vaccines are safe" and "Vaccines are effective" statements. Key: ● = country is part of the high TB burden list, ▲ = country is part of the high HIV-TB burden list, ■ = country is part of the high MDR/RR-TB burden list. For the detailed data table please see Additional File 1: Table S1.

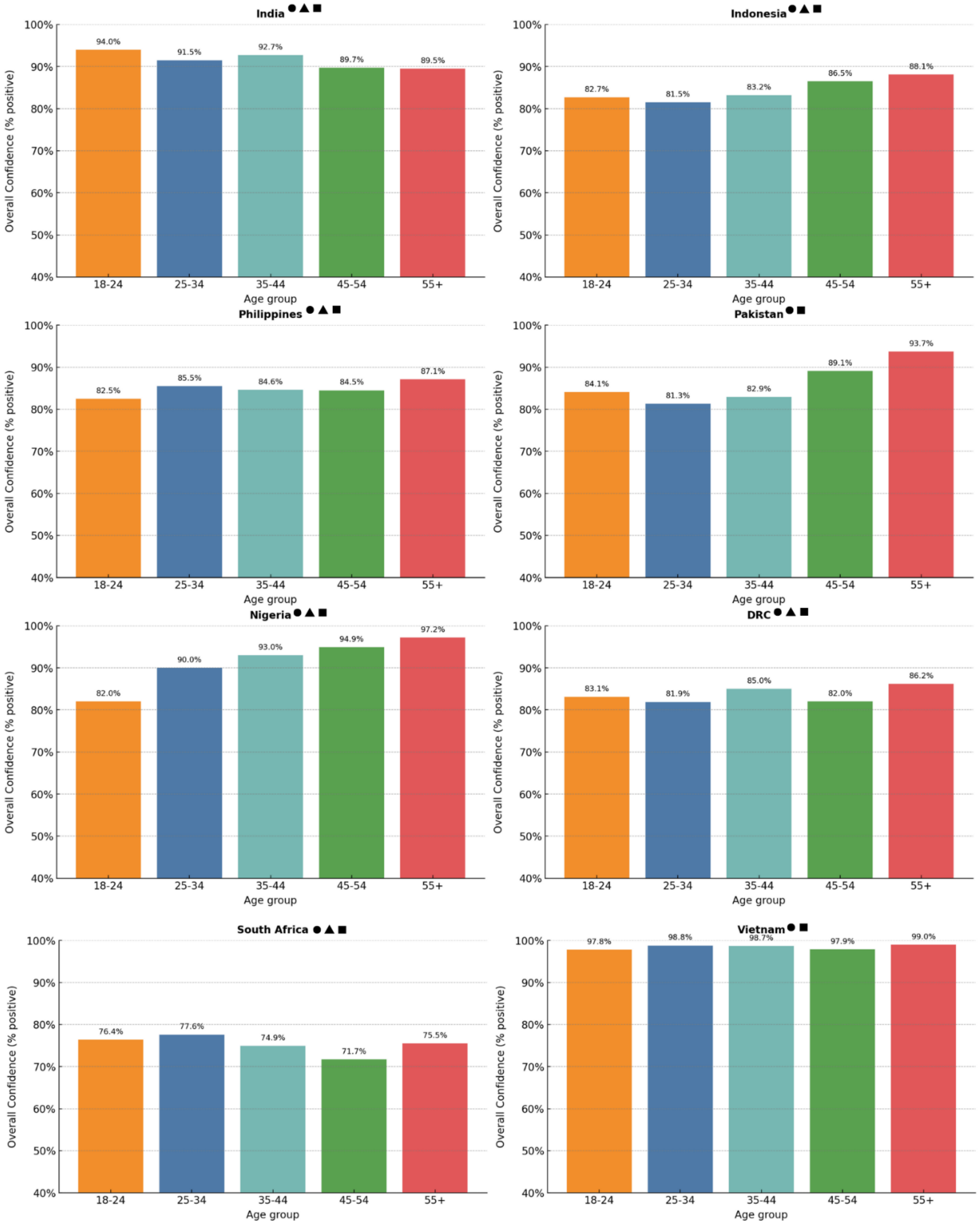
India
94.0%
91.5%
92.7%
89.7%
89.5%
Indonesia
82.7%
81.5%
83.2%
86.5%
88.1%
Philippines
82.5%
85.5%
84.6%
84.5%
87.1%
Pakistan
84.1%
81.3%
82.9%
89.1%
93.7%
Nigeria
82.0%
90.0%
93.0%
94.9%
97.2%
DRC
83.1%
81.9%
85.0%
82.0%
86.2%
South Africa
76.4%
77.6%
74.9%
71.7%
75.5%
Vietnam
97.8%
98.8%
98.7%
97.9%
99.0%
Overall Confidence (% positive)
Age group
18-24
25-34
35-44
45-54
55+
40%
50%
60%
70%
80%
90%
100%

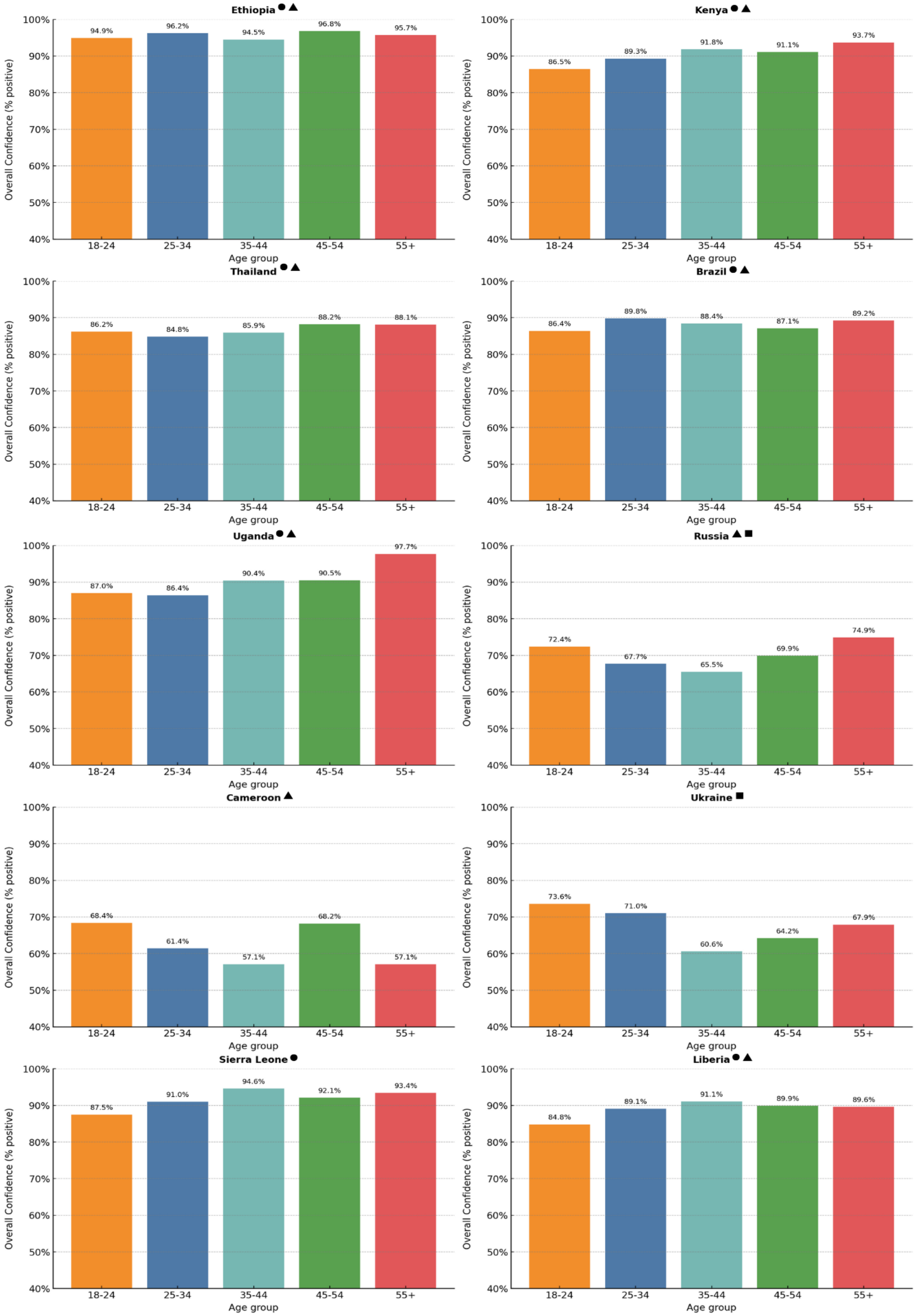

Ethiopia
94.9%
96.2%
94.5%
96.8%
95.7%
Kenya
86.5%
89.3%
91.8%
91.1%
93.7%
Thailand
86.2%
84.8%
85.9%
88.2%
88.1%
Brazil
86.4%
89.8%
88.4%
87.1%
89.2%
Uganda
87.0%
86.4%
90.4%
90.5%
97.7%
Russia
72.4%
67.7%
65.5%
69.9%
74.9%
Cameroon
68.4%
61.4%
57.1%
68.2%
57.1%
Ukraine
73.6%
71.0%
60.6%
64.2%
67.9%
Sierra Leone
87.5%
91.0%
94.6%
92.1%
93.4%
Liberia
84.8%
89.1%
91.1%
89.9%
89.6%
Overall Confidence (% positive)
100%
90%
80%
70%
60%
50%
40%
18-24
25-34
35-44
45-54
55+
Age group

Figure S2. Age-disaggregated overall vaccine confidence score by country. For each of the 18 high TB burden countries surveyed in 2023, five bar charts representing the age-group vaccine confidence scores (for age groups 18-24, 25-34, 35-44, 45-54, 55+) calculated as the average % positive responses to the three statements "Vaccines are important", "Vaccines are safe" and "Vaccines are effective" statements. Key: ● = country is part of the high TB burden list, ▲ = country is part of the high HIV-TB burden list, ■ = country is part of the high MDR/RR-TB burden list. For the detailed data table please see Additional File 1: Table S2.
